# PR55α-controlled protein phosphatase 2A inhibits p16 expression and blocks cellular senescence induction by γ-irradiation

**DOI:** 10.18632/aging.205619

**Published:** 2024-03-04

**Authors:** Chitra Palanivel, Lepakshe S. V. Madduri, Ashley L. Hein, Christopher B. Jenkins, Brendan T. Graff, Alison L. Camero, Sumin Zhou, Charles A. Enke, Michel M. Ouellette, Ying Yan

**Affiliations:** 1 Department of Radiation Oncology, University of Nebraska Medical Center, Omaha, NE 68198, USA; 2 Department of Pathology and Microbiology, University of Nebraska Medical Center, Omaha, NE 68198, USA; 3 Department of Genetics, Cell Biology, and Anatomy, University of Nebraska Medical Center, Omaha, NE 68198, USA; 4 Department of Internal Medicine - Gastroenterology and Hepatology, University of Nebraska Medical Center, Omaha, NE 68198, USA; 5 Department of Biochemistry and Molecular Biology, University of Nebraska Medical Center, Omaha, NE 68198, USA

**Keywords:** p16, p14, CDKN2A locus, p53, RB, PR55α, PP2A, γ-irradiation

## Abstract

Cellular senescence is a permanent cell cycle arrest that can be triggered by both internal and external genotoxic stressors, such as telomere dysfunction and DNA damage. The execution of senescence is mainly by two pathways, p16/RB and p53/p21, which lead to CDK4/6 inhibition and RB activation to block cell cycle progression. While the regulation of p53/p21 signaling in response to DNA damage and other insults is well-defined, the regulation of the p16/RB pathway in response to various stressors remains poorly understood. Here, we report a novel function of PR55α, a regulatory subunit of PP2A Ser/Thr phosphatase, as a potent inhibitor of p16 expression and senescence induction by ionizing radiation (IR), such as γ-rays. The results show that ectopic PR55α expression in normal pancreatic cells inhibits p16 transcription, increases RB phosphorylation, and blocks IR-induced senescence. Conversely, PR55α-knockdown by shRNA in pancreatic cancer cells elevates p16 transcription, reduces RB phosphorylation, and triggers senescence induction after IR. Furthermore, this PR55α function in the regulation of p16 and senescence is p53-independent because it was unaffected by the mutational status of p53. Moreover, PR55α only affects p16 expression but not p14 (ARF) expression, which is also transcribed from the same *CDKN2A* locus but from an alternative promoter. In normal human tissues, levels of p16 and PR55α proteins were inversely correlated and mutually exclusive. Collectively, these results describe a novel function of PR55α/PP2A in blocking p16/RB signaling and IR-induced cellular senescence.

## INTRODUCTION

**Figure 1:**
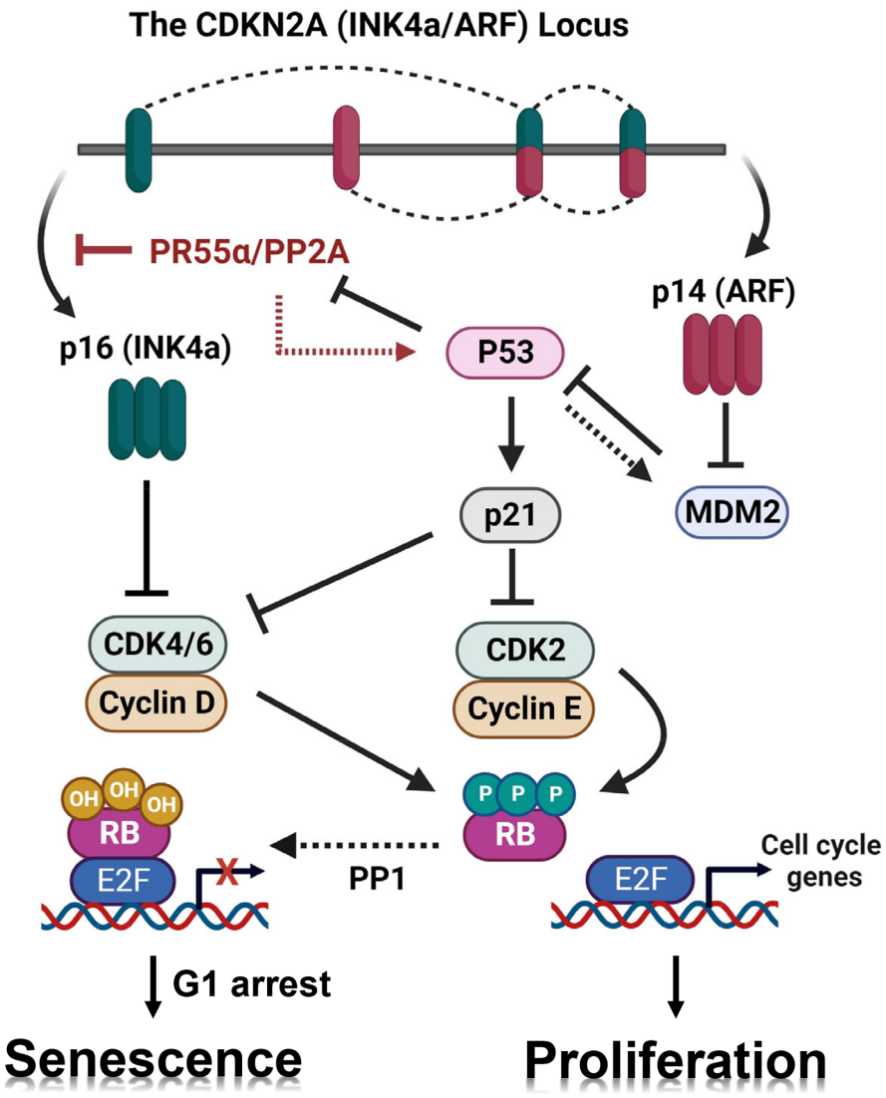
**Working model for the regulation of the p16/RB pathway and senescence induction by PR55α.** Black lines depict our current understanding of the respective roles of the p16/RB and p53/p21 pathways in the promotion of cellular senescence in response to genotoxic stressors [83, 84]. The *CDKN2A* locus produces both the p16 (INK4a) and p14 (ARF) proteins using both separate promoters and alternative splicing. The p16 protein blocks the CDK4/6 kinases leading to RB activation, which is required for G1 cell cycle arrest and senescence induction. The p14 protein stabilizes p53 by inhibiting the MDM2 E3 ubiquitin ligase, resulting in p21 (a p53 target gene) induction and subsequent inhibition of CDK2 and CDK4/6, which also leads to RB activation that promotes G1 cell cycle arrest and senescence. We have previously reported that p53 negatively regulates PR55α protein stability [28]. However, the p53 mutational status had no detectable impact on the effects of PR55α on the expression of p16 and induction of senescence by IR. Red lines depict novel findings presented in this report: (1) PR55α-controlled PP2A enhances IR-induced p53/p21 signaling and (2) PR55α inhibits p16 transcription independently of p53 function.

Senescence in normal cells is a permanent cell cycle arrest that can be triggered in response to various intrinsic and extrinsic genotoxic stressors, such as telomere dysfunction, oxidative stress, and DNA damage [1, 2]. Irrespective of the stimulus, senescent cells become permanently arrested in the G1 phase and this arrest is accompanied by alterations in phenotypic properties and gene expression. Among these changes, the induction of senescence-associated β-galactosidase (SA-β-gal) activity is the hallmark of senescent cells [3]. Senescence induction is mainly executed by one of two distinct pathways or by both: the p16 pathway that inhibits the CDK4/6 kinases and the p53/p21 pathway that inhibits the CDK2 kinase [4–6] (Figure 1). Both CDK4/6 and CDK2 activities are essential for cells to progress through the G1/S transition of the cell cycle and initiate DNA replication in the S-phase [7]. The inhibition of CDKs by p16 and/or p21 results in the hypo-phosphorylation and activation of the RB protein, which subsequently blocks cell cycle progression. While in some circumstances there is crosstalk between the p53/p21 and p16 pathways, the two pathways operate independently and can be activated separately [8, 9].

The expression of p16 and p21 is primarily regulated at the mRNA level. The up-regulation of p21 observed at senescence is induced by p53 [10], whereas the up-regulation of p16 is more complex and involves multiple mechanisms, most of which remain poorly understood. The p16 protein is encoded by the *CDKN2A* locus, which also encodes for the p14 protein (also known as ARF), an upstream activator of p53. In young and unstressed cells, the *CDKN2A* locus is maintained in an epigenetically repressed state by PcG protein complexes and the repressive histone marks that they generate (H3K27me3, H2AK119Ub) [11]. The expression of p16 requires alterations in PcG function and recruitment of transcription factors and other epigenetic regulators, such as the DDB1/CUL4-MML1 complex. In addition, p16 mRNA expression can be regulated at the post-transcriptional level. When cells undergo replicative senescence, the p16 mRNA becomes stabilized and this requires elements located in its 3′-untranslated region (3′-UTR) and 5′-UTR [12, 13]. The AU-rich element-binding factor 1 (AUF1), also called heterogeneous nuclear ribonucleoprotein D (hnRNP D), is a well-characterized RNA-binding protein that recognizes adenylate/uridylate-rich elements known to control the stability of mRNA transcripts [14]. The AUF1/hnRNP D locus (hereafter referred to as AUF1) produces transcripts encoding four isoforms (p37, p40, p42, and p45), which differ slightly in their RNA-binding abilities [15]. With other subunits, AUF1 is part of a large complex that destabilizes mRNAs carrying AU-rich elements in their 5′-UTR or 3′-UTR, as in the case of the p16 mRNA, where the binding of AUF1 leads to rapid mRNA decay [16].

The *CDKN2A* locus on chromosome 9p21, a region that encodes both p16 and p14, is frequently deleted or otherwise altered in cancers. The p16 and p14 mRNAs are both made of three exons, the second and third of which are shared between the two [17]. To produce two entirely different proteins from the same locus, the two mRNA are transcribed from separate promoters and read their common exons in different frames (Figure 1). Functionally, p16 inhibits CDK4/6, which then blocks the G1/S transition by preventing the phosphorylation and inactivation of RB, while p14 activates p53 by inhibiting MDM2, which acts as an E3 ubiquitin ligase that targets p53 for degradation [18]. At senescence, the p16 and p14 proteins can be induced together or just p16 alone, depending on the conditions, the cell type, or the species. Through their respective regulation of the RB and p53 pathways, p16 and p14 proteins serve as key regulators of both the cell cycle and cellular senescence [19].

Protein phosphatase 2A (PP2A) is a family of heterotrimeric holoenzyme complexes that constitute the majority of Ser/Thr phosphatase activities in human cells [20]. Each PP2A trimer consists of a catalytic subunit, a scaffold subunit, and a regulatory subunit. While both the catalytic and scaffold subunits are each produced as two highly conserved isoforms, the regulatory subunit comes in 27 different isoforms, with each dictating the substrate specificity and cellular localization of the associated holoenzyme [21]. PR55α is a PP2A regulatory subunit and PR55α-controlled PP2A complexes have been shown to regulate proteins involved in cell cycle control and response to various genotoxic stress, including DNA damage and nutrient deprivation [22]. Several reports, including ours, have shown that PR55α-controlled PP2A promotes the activation of oncogenic pathways by dephosphorylating inhibitory phosphorylation sites on key regulators of the cell cycle, including ERK1/2, β-catenin, c-Myc, and Yes-associated protein (YAP) [23–26]. Consistently, our studies have shown that PR55α supports oncogenic transformation and the malignant phenotype of pancreatic cancer cells [23, 27, 28]. In a recent report, we discovered that the protein stability of PR55α is regulated by the E3 ubiquitin ligase FBXL20 [28], a gene known to be up-regulated by the transcriptional activity of p53 [29]. Since oncogenesis and senescence are mutually exclusive events and PR55α promotes oncogenic transformation, we investigated the impacts of PR55α expression on the induction of premature senescence by ionizing radiation (IR). The results of this study reveal an essential role for PR55α-controlled PP2A as a potent inhibitor of p16 expression and induction of senescence by IR. The data also indicate that this function of PR55α is p53 independent since it is also observed in p53 mutant cancer cells.

## RESULTS

### PR55α inhibits the expression of p16 in human pancreatic normal and cancer cells

The p16 tumor suppressor is a major CDK4/6 inhibitor that plays an essential role in the induction of cellular senescence [30]. The loss of p16 occurs in >90% of pancreatic cancer and has been identified as a driver mutation in pancreatic cancer tumorigenesis [31]. We have previously reported an essential role for PR55α in maintaining the tumorigenicity and metastatic potential of human pancreatic cancer cells [23, 27]. Here, we assessed the impacts of PR55α on the expression and function of p16 in both HPNE cells and CD18/HPAF cells. HPNE cells are human normal pancreatic ductal cells that we previously immortalized with telomerase [32, 33]. CD18/HPAF cells are human pancreatic cancer cells driven by the KRAS oncogene and mutant p53^P151S^ [34].

Through retroviral transductions, stable lines of normal HPNE cells were engineered to express a Dox-inducible PR55α, while stable lines of CD18/HPAF cells were engineered to express a Dox-inducible PR55α-shRNA. Since p16 plays an essential role in most forms of cellular senescence, from replicative senescence to stress-induced premature senescence, we assessed the effects of PR55α on p16 expression and premature senescence-induced by IR in the context of both normal cells and cancer cells of the pancreas. To induce senescence in these experiments, we have used a high dose of radiation (10 Gy), similar to doses used in extreme hypofractionation radiation therapy, now made possible by advances in stereotactic radiotherapy [35, 36]. In radioresistant forms of cancer, such as pancreatic cancer, this approach may be producing better outcomes.

**Figure 2:**
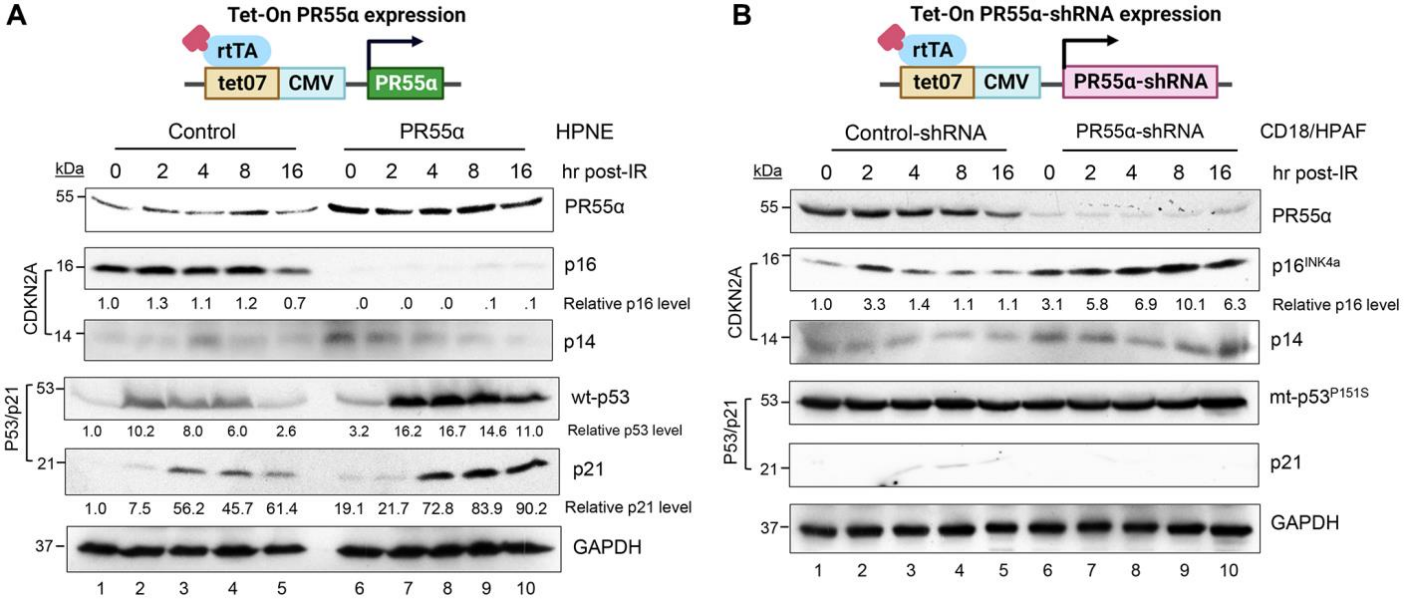
**PR55α suppresses p16 protein expression in normal and cancer cells originating from the human exocrine pancreas.** (**A**) Human pancreatic normal ductal (HPNE) cells were stably transduced with a retroviral vector expressing a Dox-inducible PR55α cDNA (PR55α). As a control, HPNE cells stably transduced with a relevant empty retroviral vector were included in the analysis (Control). Following the induction of ectopic PR55α expression by Dox (1 μg/ml) for 2 days, cells were exposed to 10 Gy of ionizing radiation (IR), incubated for the indicated hours, and analyzed by immunoblotting for the levels of p16, p14, p53, and p21. GAPDH in the lysates was measured as an internal control. (**B**) Human pancreatic ductal adenocarcinoma cells (CD18/HPAF) were stably transduced with a lentiviral vector expressing a Dox-inducible shRNA against either PR55α (PR55α-shRNA) or an irrelevant negative control (Control-shRNA). Following induction of the shRNA with Dox (2 μg/ml) for 5 days, the cells were exposed to 10 Gy IR, incubated for the indicated hours, and analyzed by immunoblotting for the levels of the indicated proteins. GAPDH in the lysates was again used as an internal control. The difference in the p16 levels between HPNE-Control and HPNE-PR55α cells, as well as between the CD18/HPAF-Control-shRNA and CD18/HPAF-PR55α-shRNA cells were determined to be statistically significant (HPNE-Control *vs.* HPNE-PR55α, *p* < 0.001; CD18/HPAF-Control-shRNA vs. CD18/HPAF-PR55α-shRNA, *p* = 0.004).

In the normal HPNE cells, PR55α induction abolished the expression of p16 (Figure 2A). After exposure to γ-irradiation, p16 expression was slightly increased in both the control and PR55α-overexpressing HPNE cells. However, the magnitude of the minor increase of p16 induced by IR was the same in the control cells and PR55α-overexpressing cells (Figure 2A). For validation, we tested the effect of PR55α on p16 expression in CD18/HPAF pancreatic cancer cells, which harbor a p53^P152S^ mutant thus lacking a functional p53/p21 pathway [34]. Although CD18/HPAF cells express a p16 protein that contains an in-frame deletion from amino acids (aa) 29 to 34 [37], this mutation does not appear to affect p16 mRNA stability, protein expression, or even p16 function since it does not interfere with the ability of p16 to bind and inhibit CDK4/6, a function mediated by its Ankyrin repeats AR2 (aa 36–69) and AR3 (aa 70–100) [38]. As predicted, the knockdown of PR55α by shRNA in CD18/HPAF cells resulted in the opposite effect compared to the overexpression of PR55α in the normal HPNE cells. Again, the high levels of PR55α expression were associated with low levels of p16 (Figure 2B). IR exposure also resulted in a subtle increase in p16 levels in CD18/HPAF cells over time, but the magnitude of this induction was the same in the control and PR55α-knockdown cells (Figure 2B). It was also noticed that there was a transient increase of the p16 level in control cells at 2 hours following IR. The cause of the effect is unknown, which could be due to a temporary increase in the protein stability of p16 prompted by IR-activated stress signaling.

Because p14 and p16 are essentially transcribed from the *CDKN2A* locus, albeit from distinct promoters (Figure 1), we assessed the effects of PR55α on p14 expression after IR. In both the HPNE and CD18/HPAF cells, the manipulation of PR55α levels did not result in a similar effect on p14 as it did on p16. In the HPNE cells, for example, PR55α overexpression abolished p16 expression but it slightly increased p14 levels. These results show that PR55α specifically inhibits p16 expression but not p14 expression.

### PR55α enhances the response of the p53/p21 pathway to IR exposure

The p53/p21 pathway plays a key role in DNA damage-induced cell cycle checkpoint response and senescence induction [39]. Loss of p53 function via mutations is the most frequent event occurring across all cancer types and is a main driver of pancreatic cancer development and progression [40]. We previously reported on the role of p53 as a negative regulator of the protein stability of PR55α, whose function supports several oncogenic pathways, including ERK, β-catenin, c-Myc, and YAP [23–28]. Since p53′s role as a tumor suppressor counteracts that of PR55α in the promotion of oncogenic pathways, we examined the impacts of PR55α on the response of the p53/p21 pathway to IR.

As shown in Figure 2A, ectopic PR55α expression in HPNE cells caused a 3.2-fold elevation in the steady-state level of p53, as well as markedly enhanced p53 induction by IR in HPNE cells. Within 2 h post-IR, p53 was induced in both the HPNE/control and HPNE/PR55α cells, but the induction was 6–8 fold higher in the latter (Figure 2A). To validate these results, we assessed the effects of PR55α on p21 expression, which is known to be induced by the transcriptional activity of p53 [41]. Consistently, ectopic PR55α expression in HPNE cells led to higher steady-state levels of p21, which is in line with its effect on p53 (Figure 2A). Like p53 levels, p21 levels were also induced by IR, albeit with a kinetic that was delayed by 2 hours compared to the induction of p53 (Figure 2A). Similar to p53, the magnitude of the induction of p21 by IR was higher in HPNE/PR55α cells compared to the HPNE/control cells (Figure 2A).

For comparison, we assessed the effect of PR55α level on mutant p53 expression in CD18/HPAF pancreatic cancer cells. The mutant p53 expressed in CD18/HPAF cells harbors a P151S mutation in the DNA binding domain of p53, thus abolishing its activity as a transcription factor [34]. As shown in Figure 2B, the knockdown of PR55α by shRNA did not affect the level of the p53^P151S^ mutant protein, either in the presence or absence of IR (Figure 2B), which was in contrast to the situation observed in HPNE cells known to express wild-type (wt) p53 protein (Figure 2A). In agreement with the lack of functional p53 in CD18/HPAF cells, levels of p21 were extremely low in these cells and were not particularly affected by either the knockdown of PR55α or exposure to IR (Figure 2B). These results indicate that PR55α only increases the expression of wt-p53, but not mutant p53. Taken together with our analyses of *CDKN2A* gene products, these results show that PR55α inhibits p16 expression while simultaneously enhancing the expression of wt p53. However, this enhancement of p53 expression is not associated with changes in p14 levels and is thus independent of p14.

### PR55α suppresses p16 expression by inhibiting its mRNA transcription

**Figure 3:**
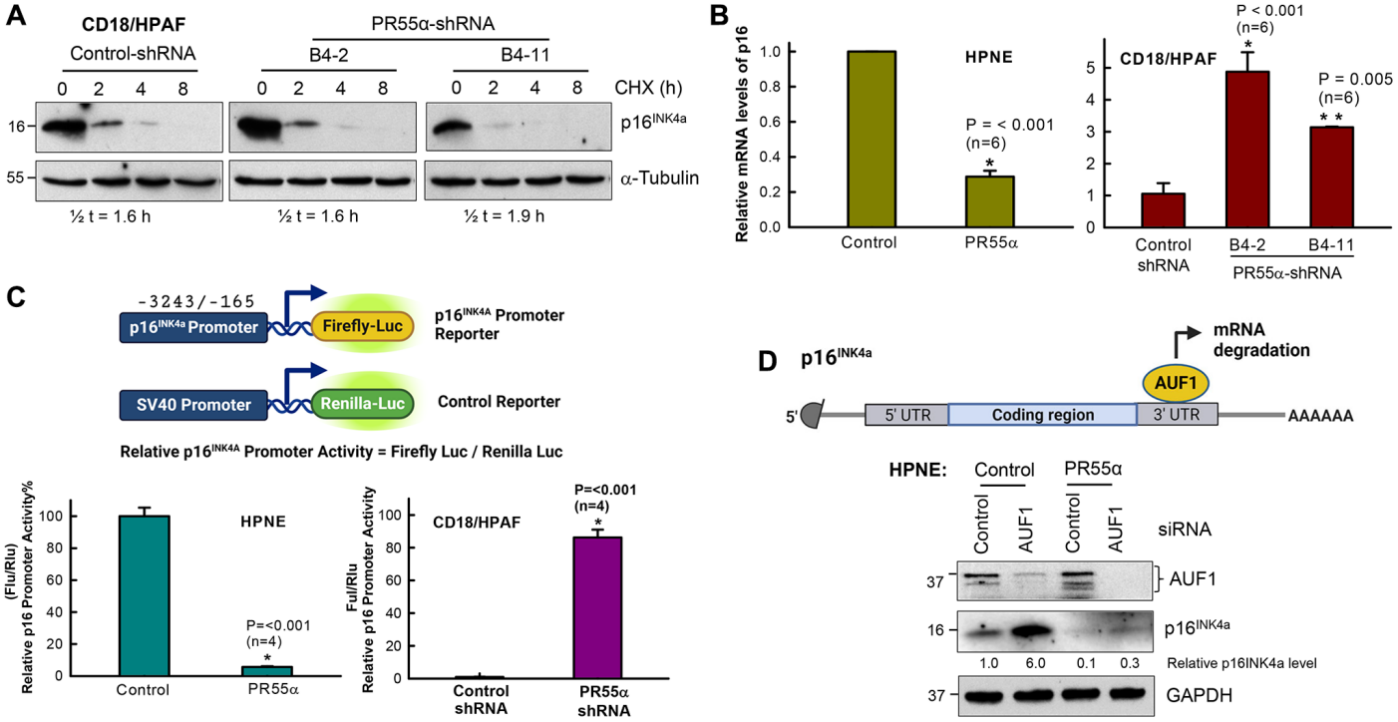
**PR55α inhibits p16 expression by suppressing its mRNA transcription.** (**A**) PR55α does not affect p16 protein stability. CD18/HPAF cells stably transduced with Dox-inducible PR55α-shRNA or Control-shRNA were cultivated in media containing 2 μg/ml Dox for 5 days to induce the shRNA, after which cells were exposed to cycloheximide (CHX, 15 μg/ml) to halt protein synthesis. Cell lysates collected at the indicated time points after CHX addition were analyzed for changes in p16 protein levels. The α-tubulin protein has a long half-life and was used as an internal control. Relative p16 protein levels were determined after normalization with the α-tubulin levels and these normalized values were used to calculate the protein half-life of p16. Half-lives were estimated by linear regression analysis of p16 normalized levels against time using SigmaPlot. (**B**) PR55α inhibits p16 mRNA expression. Left panel: HPNE expressing the Dox-inducible PR55α (HPNE/PR55α), or empty vector (HPNE/Control) was treated with Dox (1 μg/ml) for 3 days. Right panel: CD18/HPAF cells expressing the Dox-inducible PR55α shRNA (B4-2, B4-11) or Control shRNA (Control) were treated with Dox (2 μg/ml) for 5 days. At the end of each treatment, RNA was isolated and analyzed by quantitative RT-PCR for differences in p16 mRNA. The relative abundance of the p16 mRNA was calculated by normalizing the p16 mRNA levels with those of the GAPDH mRNA, with the data represented as mean ± S.D. (bar graphs). Statistical significance was calculated by a Student’s *t*-test (HPNE cells) or one-way ANOVA (CD18/HPAF). The difference with the Control group (*n* = 6/group) was determined to be statistically significant at ^*^p < 0.001 or ^**^p < 0.005. (**C**) PR55α suppresses p16 promoter activity. HPNE and CD18/HPAF cells in the presence/absence of ectopic PR55α and PR55α-shRNA expression, respectively, were co-transfected with a Firefly luciferase reporter under the control of the p16 promoter and a control Renilla luciferase reporter driven by the SV40 promoter. Two days after transfection, Firefly, and Renilla luciferase activities were measured separately in each lysate, as described in the Materials and Methods. p16 promoter activity was calculated by normalizing the activity of Firefly luciferase over that of Renilla luciferase. The graphs show relative p16 promoter activities in the indicated cell samples and are expressed as the mean ± S.D. of two independent experiments done in duplicates. ^*^Statistically significant in a Student’s *t*-test with *p* < 0.001. (**D**) HPNE/Control and HPNE/PR55α cells were incubated in the presence of 1 μg/ml Dox for 48 h to induce PR55α expression, after which cells were transfected with either a non-targeting siRNA (*Control*) or AUF1 siRNA. Two days later, cells were analyzed by immunoblotting for differences in levels of AUF1 and p16. GAPDH was used as an internal standard. The levels of p16 and GAPDH were quantified using Fiji-ImageJ software and relative p16 levels in the samples were determined after normalizing it with GAPDH levels.

To define the mechanism by which PR55α inhibits p16 expression, we first examined the impacts of PR55α on the protein stability of p16 using the cycloheximide (CHX)-chase assay, as described in our previous work [28]. CD18/HPAF cells expressing the control- or PR55α-shRNA were incubated with 15 μg/ml cycloheximide to block protein synthesis to allow measurements of p16 protein decay. As depicted in Figure 3A, PR55α knockdown by shRNA did not result in a significant change in the p16 protein half-life in CD18/HPAF cells, indicating a lack of effect of PR55α on the p16 protein decay process.

We next assessed the effects of PR55α on p16 mRNA expression using real-time quantitative Reverse Transcription and PCR (qRT-PCR). As shown in Figure 3B, the p16 mRNA level was decreased by ~70% in the HPNE cells expressing ectopic PR55α compared to the control HPNE cells. Conversely, the PR55α knockdown by shRNA resulted in a 3–5 fold induction in p16 mRNA level in CD18/HPAF cells compared to the control cells (Figure 3B). Statistically, these changes were all determined to be highly significant. To further define the mechanism by which PR55α reduces p16 mRNA expression, we analyzed the impact of PR55α on p16 promoter activity using a luciferase reporter assay. HPNE and CD18/HPAF cells were transiently co-transfected with two reporter vectors: a vector expressing firefly luciferase under the control of the p16 promoter (nucleotides −3243 to −165) and a control vector expressing Renilla luciferase from the SV40 promoter (Figure 3C). As shown in Figure 3C, ectopic PR55α expression in HPNE normal cells resulted in >90% inhibition of the p16 promoter activity compared to the control cells. Conversely, shRNA knockdown of PR55α in CD18/HPAF cancer cells resulted in approximately 85-fold induction in the p16 promoter activity. Together, these results indicate that the inhibition of p16 expression by PR55α is at least in part mediated by the repression of its promoter activity.

### Knocking down AUF1 does not affect the inhibition of p16 expression by PR55α

AUF1 is an RNA-binding protein that has been reported to promote p16 mRNA decay during replicative senescence [13]. Thus, we examined the effect of AUF1 on the suppression of p16 expression by PR55α. AUF1 was knocked down by siRNA in both HPNE control and PR55α overexpressing cells and the resulting cells were analyzed for differences in the levels of AUF1 and p16 after 48 h incubation. In HPNE control cells, the knockdown of AUF1 resulted in a 6-fold induction of p16 protein expression (Figure 3D), which is consistent with previous findings [13]. However, in the PR55α overexpressing cells, the knockdown of AUF1 did not suffice to reallow the expression of the p16 protein (Figure 3D). Thus, the AUF1-mediated mRNA decay mechanism is not responsible for the inhibition of p16 expression by PR55α.

### PR55α promotes RB phosphorylation

RB plays a key role in the induction of both replicative senescence and stress-induced premature senescence, both of which are known to exhibit a permanent cell cycle arrest at the G1 phase caused by the sequestration of the E2F transcription factor by RB [42–44]. During cell proliferation, RB is phosphorylated by CDK4/6 and then CDK2 kinases, which free E2F to induce the transcription of genes needed for the S phase [45]. In senescent cells, CDK4/6 and CDK2 are permanently inhibited by the induction of the p16 and p21 proteins, respectively [46] (Figure 1). Since PR55α affected the expression of both p16 and p21 but in different ways (Figure 2), we investigated the net effect of PR55α on RB phosphorylation.

**Figure 4:**
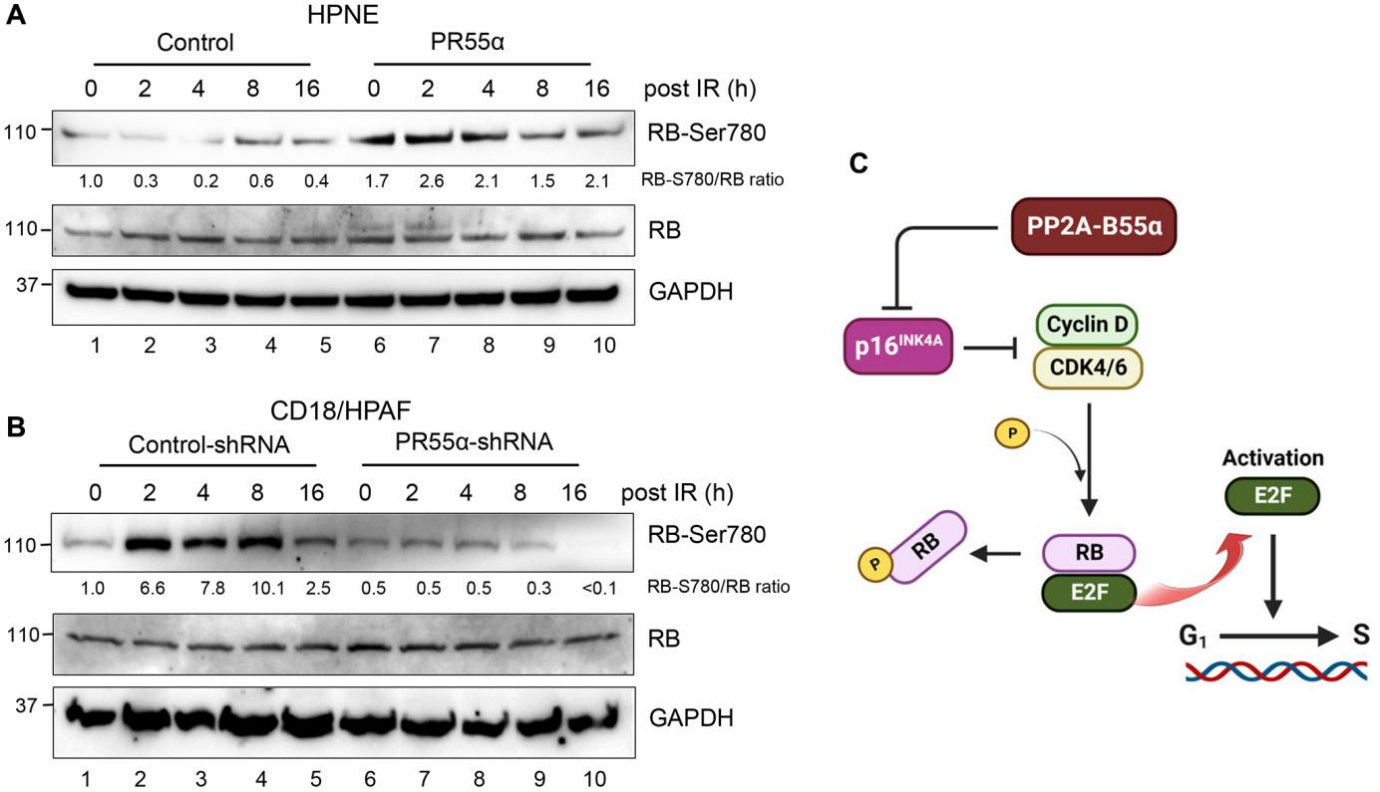
**PR55α expression increases RB phosphorylation in normal and malignant pancreatic cells.** (**A**) A schematic depiction of the regulation of the p16/RB cascade by PR55α-controlled PP2A enzymes. PR55α suppresses the expression of p16, which acts as the primary inhibitor of the CDK4/6 kinases that phosphorylate and inactivate RB, thereby resulting in its dissociation from the E2F transcriptional factor. The net effect of PR55α is promoting the G1/S transition by allowing the phosphorylation of RB and the release of E2F. (**B**) HPNE cells transduced with Dox-inducible PR55α, or control vector were incubated with 1 μg/ml Dox for 3 days to induce ectopic PR55α expression. The cells were exposed to 10 Gy IR and then incubated for the times indicated. Harvested cells were analyzed for RB-Ser708 phosphorylation, total RB level, and GAPDH (as an internal control). (**C**) CD18/HPAF-transduced with the Control or PR55α shRNA were incubated with 2 μg/ml Dox for 5 days to knockdown PR55α expression, after which cells were analyzed by immunoblotting for differences in RB-Ser708 phosphorylation, total RB, and GAPDH.

Consistent with the preponderant role played by p16 in cellular senescence [47], the effects of PR55α on RB phosphorylation mirrored those observed with the p16 protein in both the normal and cancer cells. In HPNE cells, where PR55α overexpression suppresses p16 expression (Figure 2A, lane 6 vs. 1), the phosphorylation of RB-Ser780 (Figure 4A, lane 6 vs. 1) was elevated, indicative of increased CDK4/6 kinase activity [48, 49]. Similar to the situation observed with p16 in the HPNE/PR55α cells (Figure 2A), IR only had minor impacts on the Ser780 phosphorylation of RB, which remained high across all time points post-IR (Figure 4A).

For validation, we examined the effect of PR55α on RB phosphorylation in CD18/HPAF pancreatic cancer cells. The knockdown of PR55α resulted in a ~50% decrease in the steady-state phosphorylation of RB-Ser780 in the log-phase CD18/HPAF cells (Figure 4B, lane 6 vs. 1), which is consistent with the elevated p16 levels in these cells relative to the control cells (Figure 2B, lane 6 vs. 1). Furthermore, IR exposure of control CD18/HPAF cells resulted in the induction in RB-Ser780 phosphorylation, whereas IR exposure of PR55α-knockdown CD18/HPAF cells rather resulted in a time-dependent decrease in RB-Ser780 phosphorylation (Figure 4B). Together, these results support a role for PR55α in the suppression of p16/RB signaling (Figure 4C).

### PR55α impedes the cell cycle response of pancreatic normal and cancer cells to IR exposure

In response to DNA damage, cells elicit a cell cycle checkpoint response, leading to either cell cycle arrest for DNA repair, or replicative cell death (apoptosis or else senescence) if the damage is irreversible [50, 51]. Normal cells possess wild-type p53, which can be rapidly activated by DNA damage to induce p21 expression and arrest the cell cycle at the G1 phase while promoting DNA repair [52]. However, most cancer cells harbor p53 mutations and are thus defective in the G1 checkpoint that requires the p53/p21 pathway. In these cancer cells, DNA damage only activates the G2 checkpoint, thereby blocking the G2/M transition to allow DNA repair [53]. Yet, both the G1 and G2 checkpoints are dependent on the activation of RB to block cell cycle progression and this activation can be triggered by either the p53/p21 pathway and/or the p16/RB pathway (Figure 1) [8]. Because PR55α inhibits p16 expression in both the normal HPNE cells expressing wild-type p53 and the CD18/HPAF cancer cells expressing mutant p53, we decided to examine the impacts of PR55α on the IR-induced cell cycle response. For this purpose, exponentially proliferating HPNE and CD18/HPAF cells, with/without PR55α manipulations, were exposed to increased doses of IR and analyzed 24 hours later for changes in DNA content by Fluorescence-activated cell sorting (FACS).

**Figure 5:**
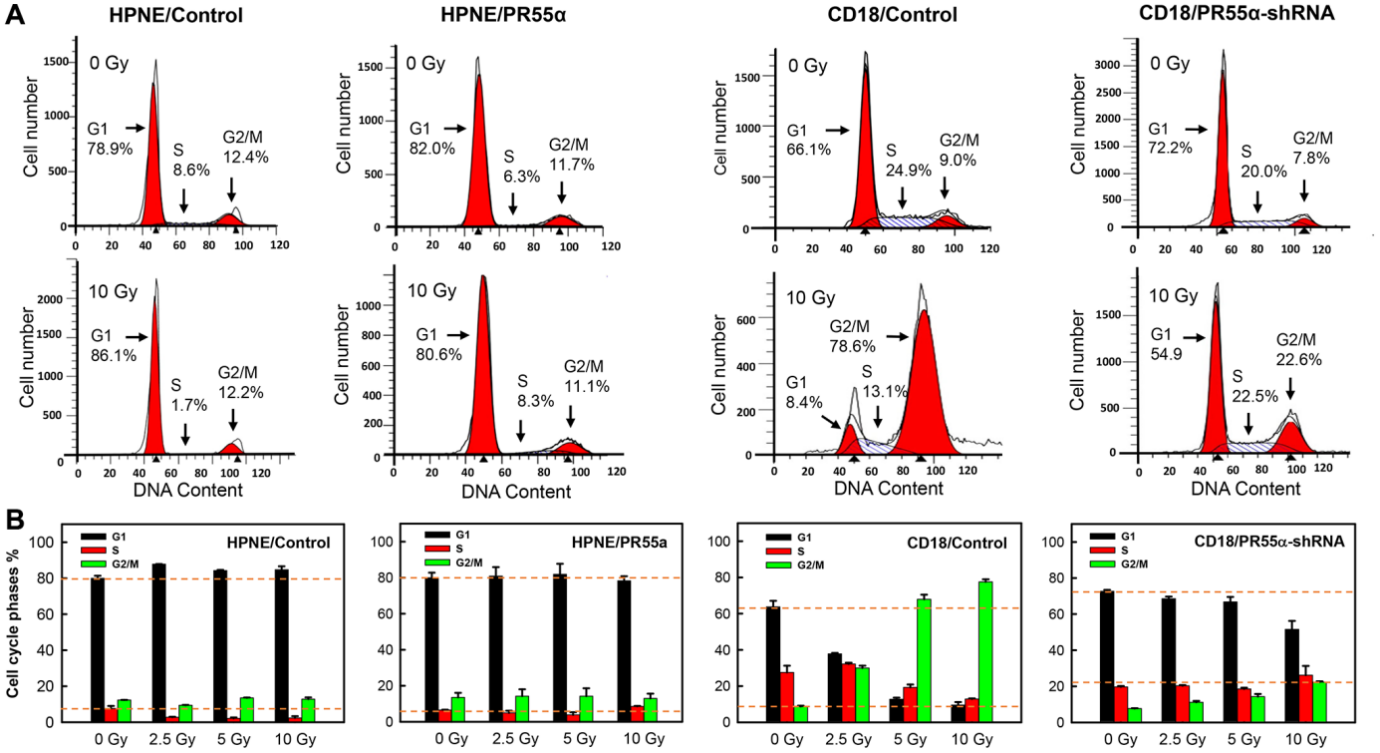
**Effect of PR55α on the cell cycle response of normal and cancer cells to IR.** Log-phase growing HPNE (Control- or PR55α-transduced) and CD18/HPAF cells (Control-shRNA or PR55α-shRNA transduced) were incubated in media containing 1 μg/ml Dox for 3 days and 2 μg/ml Dox for 5 days, respectively, to manipulate PR55α expression. The cells were exposed to IR at indicated doses, incubated for 24 hours, and then stained with propidium iodide (PI) and analyzed for DNA content using FACS. (**A**) Representative FACS analyses are shown. The location and percent of cells in the G1, S, and G2/M phases of the cell cycle are indicated by arrows. (**B**) Bar graphs depicting the percent cells in the G1, S, and G2/M phases of the cell cycle. Each bar represents the mean ± S.D. of two separate experiments, each done in duplicates.

As shown in Figure 5, IR exposure of HPNE/Control cells resulted in a small but statistically significant increase in the percent cells in the G1 phase (*p* = 0.016), which was accompanied by a concurrent decrease in cells in the S phase (*p* = 0.021) and little change in the cells at the G2/M phases, all indicative of a G1 cell cycle arrest. In contrast, in PR55α-overexpressed HPNE cells (HPNE/PR55α), IR exposure produced little, if any, effects on the cell cycle profile compared to the unirradiated cells (Figure 5). Because HPNE/Control cells express both wild-type p53 and wild-type p16, their cell cycle response to IR was controlled by the combined activities of the p53/p21 and p16/RB pathways. In contrast, in HPNE/PR55α cells, since the expression of p16 was inhibited to near totality while the p53/p21 was further enhanced (Figure 2A), the effect of IR on the cell cycle response of these cells could only be attributed to the p53/p21 pathway.

Next, we examined the effect of PR55α on the cell cycle response of CD18/HPAF cells, which express the p53^P151S^ mutant along with very low levels of p16 [37, 54], making these cells deficient in both p53 and p16 functions and lacking a functional G1 cell cycle checkpoint as part of their DNA damage response [53, 55]. However, CD18/HPAF cells still possess an intact G2 checkpoint as part of their DNA damage response, which can function independently of p53 and p16 [56, 57]. As shown in Figure 5, IR exposure of CD18/HPAF control cells (*CD18/Control*) resulted in dose-dependent increases in cells at the G2/M phases that were accompanied by concurrent diminutions in percent cells at the G1 and S phases, which is indicative of a G2/M cell cycle arrest. In the PR55α knockdown cells (*CD18/PR55α-shRNA*), while IR still led to a G2/M cell cycle arrest (Figure 5), the percent cells arrested at the G2/M phases was much reduced compared to the irradiated control cells (*CD18/Control*), by more than a factor of 3 (22.6% versus 78.6%). In the PR55α knockdown cells, IR also did not substantially deplete the percent cells in the G1 or S phases of the cell cycle. Compared to only 8.4% of CD18/Control cells that were retained in the G1 phase after IR, 54.9% of CD18/PR55α-shRNA were still detected in the G1 phase after IR, which corresponds to a 6.5-fold difference in the percent cells in the G1 phase between the two isogenic cell populations (Figure 5). Collectively, these results indicate that, although the parental CD18/HPAF cancer cells lack a functional G1 checkpoint and thus instead respond to IR with the induction of a G2/M cell cycle arrest, the knockdown of PR55α by shRNA was sufficient to induce high levels of p16 expression, which may have then allowed the bulk of the cells to remain in G1 after IR. These results from CD18/HPAF cells are again consistent with the negative regulation of p16 expression by PR55α and subsequent impacts on RB phosphorylation and cell cycle checkpoints.

### PR55α inhibits p16-dependent cellular senescence induction by IR

**Figure 6:**
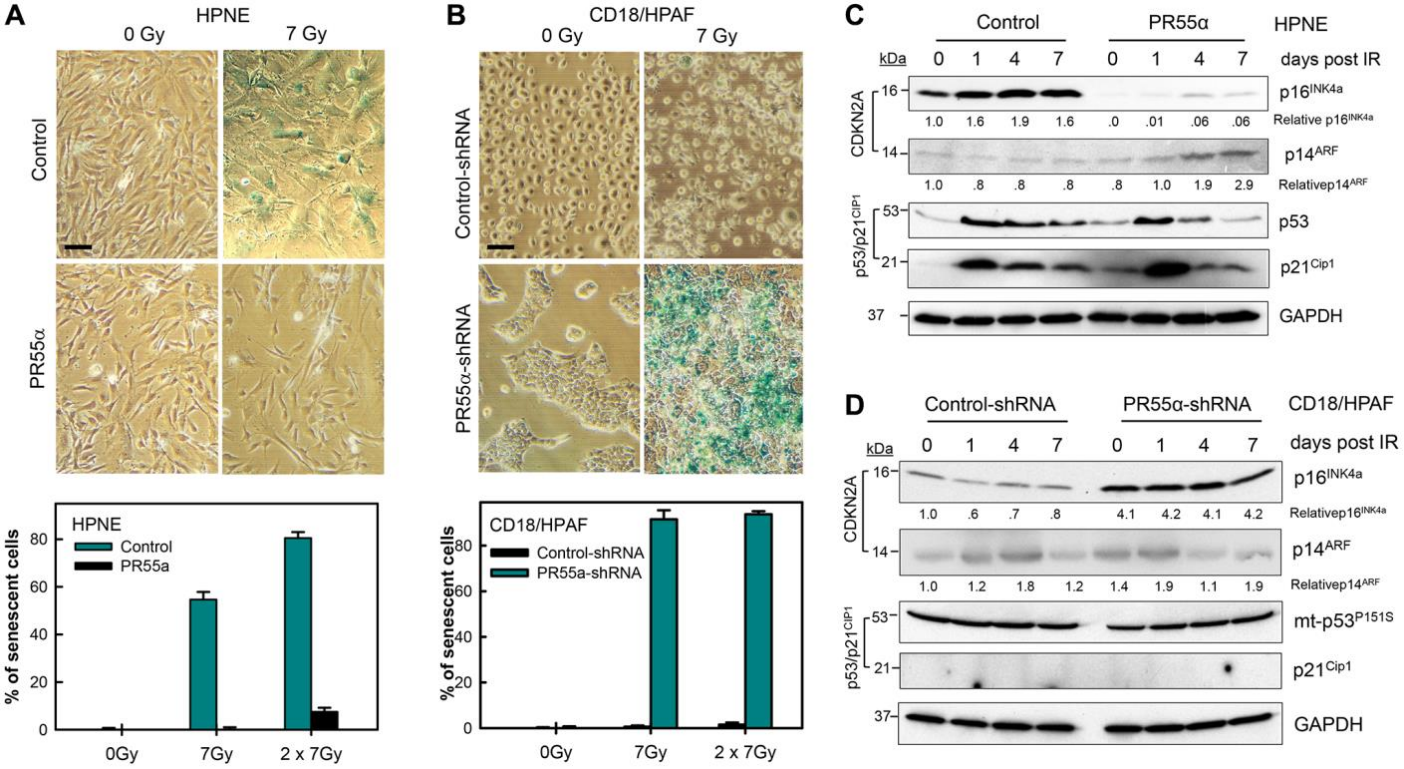
**PR55α inhibits IR-induced cellular senescence.** (**A**) Ectopic PR55α overexpression prevents senescence induction by IR in normal HPNE cells. HPNE/PR55α and HPNE/control cells were incubated in the presence of 1 μg/ml Dox for 2 days (to induce ectopic PR55α in the HPNE/PR55α cells) and then exposed to 7 Gy IR or left unirradiated as control (*0 Gy*). When the second radiation dose was applied, the interval was 24 hours between the two doses. Seven days post-IR, cells were assessed for SA-β-gal activity and photographed. Scale bar = 1 μm. The bar graph expresses the percent of senescent cells in the indicated samples and represents the mean ± S.D. of two separate experiments with each done in duplicate samples. (**B**) PR55α-knockdown sensitizes CD18/HPAF pancreatic cancer cells to senescence induction by IR. CD18/HPAF cells expressing Dox-inducible PR55α-shRNA or Control-shRNA were cultivated in the presence of 2 μg/ml Dox for 5 days, to allow time to silence PR55α expression, and then exposed to 7 Gy IR, or left unirradiated as a control (*0 Gy*). After 7 days, the cells were assessed for SA-β-gal activity and photographed. Scale bar = 1 μm. The graphs express the percent of senescent cells in the indicated samples and represent the mean ± S.D. of two separate experiments with each one in duplicate samples. (**C**, **D**) Normal HPNE and CD18/HPAF pancreatic cancer cells, with/without PR55α manipulation, were exposed to 7 Gy IR, or left unirradiated as a control (*0 day*). When the second radiation dose was applied, the interval was 24 hours between the two doses. The irradiated cells were incubated for the times indicated and analyzed by immunoblotting for the differences in levels of p16, p14, p53, and p21. GAPDH was used as an internal control.

Our findings in Figures 2–5 reveal PR55α-controlled PP2A as a potent inhibitor of p16 expression and G1 checkpoint activation. In normal human cells, activation of the G1 checkpoint by genotoxic assaults is required for the induction of cellular senescence, both replicative and premature [7, 13, 58], to prevent the damaged cells from dividing and subsequently becoming malignant [59–61]. The induction of senescence commonly involves two key regulators: the p16/RB and/or p53/p21 pathways, depending on the insult and the cell type [4–6, 58]. To examine the effects of PR55α on senescence induction by IR, we measured the activity of senescence-associated β-galactosidase (SA-β-gal), a hallmark of senescent cells [62]. In control HPNE cells, SA-β-gal activity was undetectable before IR exposure (0 Gy) but detected in ~60% and ~80% of cells after exposure to a single (7 Gy) or double (2 × 7 Gy) doses of IR, respectively. In contrast, PR55α overexpression in HPNE cells markedly diminished senescence induction by IR, with less than 10% of irradiated HPNE/PR55α cells staining positive for SA-β-gal activity after either the 1 × 7 Gy or 2 × 7 Gy treatment (Figure 6A). Likewise, while control CD18/HPAF cells exposed to 7 Gy IR only exhibited <1% SA-β-gal positive cells at 7 days post-IR, < 90% of irradiated PR55α-knockdown CD18/HPAF cells were positive for SA-β-gal activity post-IR (Figure 6B). Senescent HPNE or CD18/HPAF cells were also typically larger and flatter in shape compared to their respective unirradiated controls (Figures 6A, 6C). Collectively, in either p53-wt HPNE normal cells or p53-mutant CD18/HPAF cancer cells, senescence induction in response to IR requires a low level of PR55α expression, thereby revealing PR55α as a potent inhibitor of IR-induced cellular senescence. Also, this regulation of p16 expression by PR55α was independent of the p53/p21 pathway, since the IR-activated p53/p21 cascade was further enhanced by the overexpression of PR55α in HPNE/PR55α cells, in which IR still failed to induce senescence. Conversely, despite CD18/HPAF cells harboring a mutant p53, the knockdown of PR55α still resulted in increased p16 expression and induction of senescence in response to IR exposure (Figures 2 and 6).

**Figure 7:**
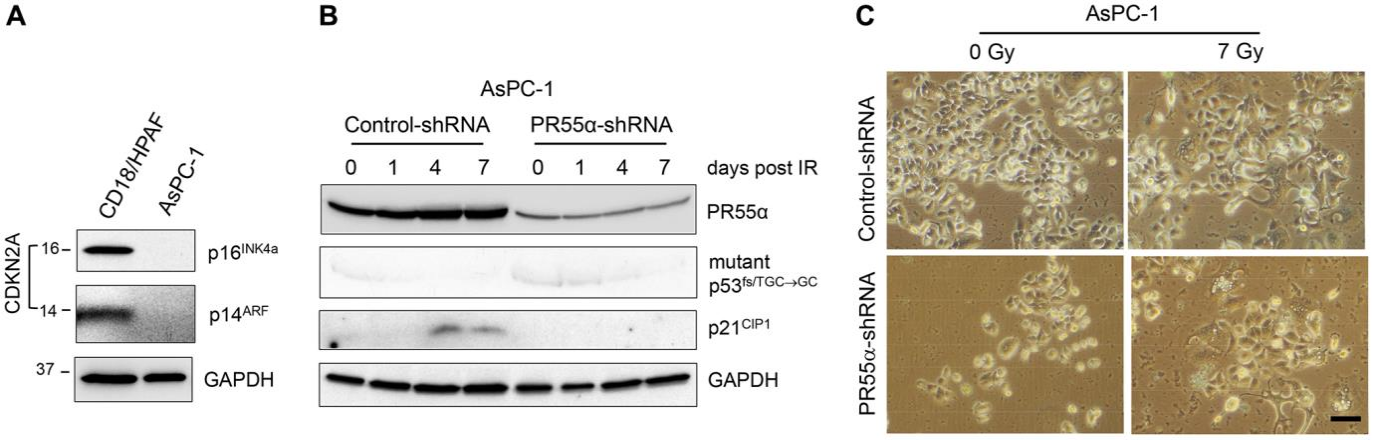
**Knockdown of PR55α does not result in senescence induction by IR in pancreatic cancer cells that lack p16 expression.** (**A**) The *CDKN2A* locus, which encodes the p16 and p14 genes, is deleted from AsPC-1 pancreatic cancer cells [37]. Log-phase growing AsPC-1 cells were analyzed for the presence of p16 and p14 proteins with CD18/HPAF pancreatic cancer cells as a positive control. (**B**) AsPC-1 cells stably transduced with the Dox-inducible PR55α-shRNA or Control-shRNA were induced with 2 μg/ml Dox for 3 days to knock down PR55α. The cells were then exposed to 7 Gy IR, or left unirradiated (0 time point), and incubated for an additional 1, 4 and 7 days. The cells were analyzed by immunoblotting for the levels of PR55α, p53, and p21. GAPDH level was measured as an internal loading control. (**C**) The irradiated cells incubated for 7 days were analyzed for senescence by SA-β-gal activity assay and photographed. Scale bar = 1 μm.

To confirm that p16 expression is required for senescence induction by IR in PR55α knockdown cells, we utilized the AsPC-1 pancreatic cancer cells, which carry a homologous deletion of the *CDKN2A* locus [37] and thus lack both p16 and p14 expression (Figure 7A). AsPC-1 cells express p53 from an allele that carries a single nucleotide deletion (TGC→GC) at Cys135 that produces a frameshift [37]. Hence, these cells express a truncated and dysfunctional p53 protein, which is also unstable, and are thus devoid of p21 expression (Figure 7B). Subsequently, we tested the effect of PR55α-knockdown on senescence induction by IR in AsPC-1 cells using the SA-β-galactosidase assay. As shown in Figures 7B, 7C, IR exposure of AsPC-1 cells did not lead to senescence induction either in the presence or absence of the PR55α knockdown by shRNA. These results, together with the data obtained from CD18/HPAF cells, which also express mutant p53 but possess a functional p16 (Figures 2B and 6B), suggest a role for PR55α in the inhibition of the p16/RB pathway-dependent senescence induction by IR.

### PR55α expression inversely correlates with the expression of p16 in normal human tissues

**Figure 8:**
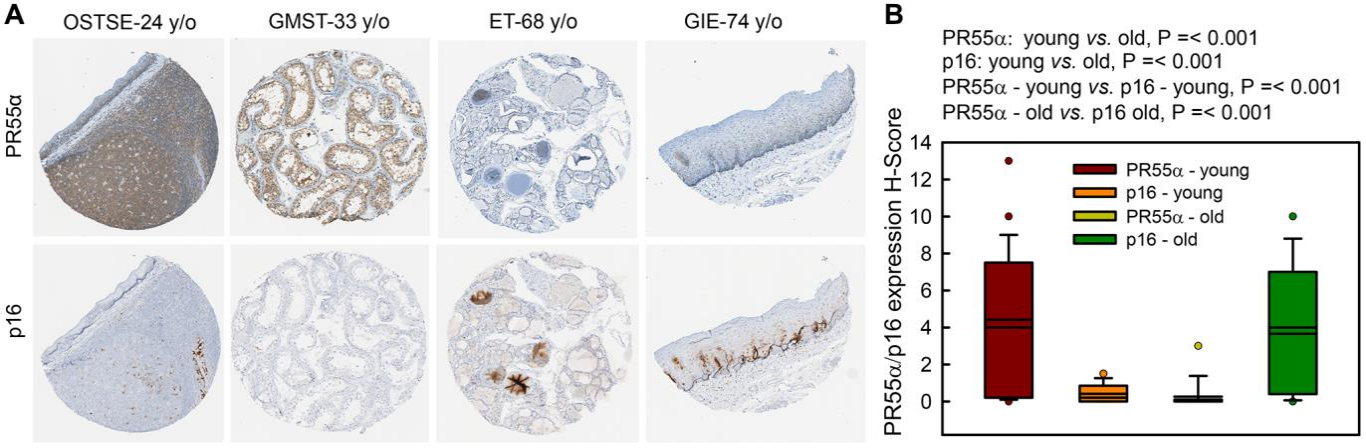
**PR55α level is much lower in human normal tissue specimens of older individuals compared to younger individuals and inversely correlates with p16 levels. Human normal tissue specimens derived from various organs/sites were analyzed for differences in PR55α and p16 expression by IHC.** (**A**) Representative images of adjacent tissue sections stained with anti-PR55α and anti-p16 antibodies. OSTSE–tonsil; GMST-seminiferous tubules; ET-thyroid; GIE-esophagus. Young, ≤43 y/o; Old, ≥68 y/o; (**B**) Box plot shows the H-Score quantification of PR55α and p16 expression from adjacent sections.

We analyzed the co-expression of PR55α and p16 in serial sections of human normal tissue specimens derived from various organs (bladder, colon, fallopian tube, heart, lung, liver, kidney, pancreas, and skin). Since p16 expression increases as a function of age, we sub-divided specimens into two cohorts based on the donor’s age: a “young” cohort consisting of tissues derived from individuals younger than 43 years old (y/o) and an “old” cohort consisting of the tissues derived from individuals older than 68 y/o. As shown in Figure 8, there was a statistically significant inverse correlation between PR55α and p16 expression in both young and old cohorts of tissues, with PR55α levels detected at higher levels in tissues of young donors compared to old donors and p16 levels exhibiting the opposite relationship. Indeed, PR55α levels were significantly lower in the old cohort compared to the young cohort. Conversely, the p16 levels were significantly higher in the old cohort compared to the young cohort.

## DISCUSSION

Cellular senescence, both replicative and stress-induced, relies on the p16/RB and/or p53/p21 pathways to inhibit the G1 CDKs to block the cell cycle at the G1/S border [42, 43, 63–65]. Acting as a major obstacle to oncogenesis, senescence is induced in response to various genotoxic stimuli, such as telomere uncapping, DNA damage, oxidative stress, and oncogene activation [66]. Consequently, the loss of function mutations in p53 and p16 tumor suppressors are the most frequent genetic alterations detected in human cancers [12, 45, 67]. In pancreatic cancer, the incidence of mutations affecting p53 and p16 function has been reported at 60–70% and 30–50% of all cases, respectively [40]. We previously reported on the essential role played by PR55α-controlled PP2A in the activation of oncogenic pathways involving ERK, YAP, β-catenin, and c-Myc, all of which are known to be essential for pancreatic cancer tumorigenesis and progression [23–26]. In this report, we present results that reveal a novel function of PR55α in the inhibition of p16 mRNA expression and abrogation of IR-induced premature senescence, and this PR55α function was observed in normal HPNE cells expressing ectopic PR55α, as well as in CD18/HPAF cells with PR55α silenced by shRNA (see Figures 2, 3, and 6). In normal HPNE cells, while ectopic PR55α inhibited p16 expression and IR-induced senescence, it simultaneously caused activation of the p53/p21 cascade (see Figure 2A). This increase in p53/p21 signaling, which did not appear to be associated with an increase in p14 (see Figures 1, 2, and 6), could be part of a negative feedback loop auto-regulating PR55α levels. In a recent article, we report that the protein stability of PR55α is negatively regulated by p53 in conjunction with the E3 ubiquitin ligase FBXL20 [28]. Hence, when PR55α is in excess, the activation of p53 could also increase the expression of the p53-regulated FBXL20 gene, thereby causing PR55α degradation. A similar loop is known to operate between p53 and its negative regulator, MDM2 [68]. Future studies will be needed to elucidate the nature of this possible auto-feedback regulatory mechanism.

A significant finding of the current study is the ability of PR55α to block cellular senescence induced by IR in both normal and cancer cells (See Figure 6). Furthermore, this novel function of PR55α is linked to its ability to inhibit the p16/RB pathway but not the p53/p21 pathway (see Figures 2 and 6), given the opposite effects of PR55α on the two pathways and the fact that PR55α regulates p16 and IR-induced senescence even in the absence of a functional p53/p21 pathway, as in CD18/HPAF cells. Conversely, in AsPC-1 pancreatic cancer cells, which contain the p16 gene homologous deletion, silencing PR55α did not result in senescence induction after IR (See Figure 7). We tested whether the re-introduction of p16 using an adenoviral vector (Ad.p16) could suffice to enable the senescence response to IR in AsPC-1 cells to allow testing the impacts of PR55α. However, ectopic p16 expression in AsPC-1 cells instead resulted in apoptosis induction, as determined by visual inspection and detection of caspase 3 cleavage (data not shown). This p16-induced apoptosis has been reported before in breast cancer cells [69] and may be due to the dependence and addiction of these cancer cells to their complete loss of endogenous p16. In summary, these results indicate that PR55α blocks senescence by specifically inhibiting p16 expression and p16/RB signaling (Figure 1).

We have previously reported that the protein stability of PR55α is negatively regulated by the p53/FBXL20 pathway [28]. We also have shown that PR55α is necessary to activate several oncogenic pathways that promote pancreatic cancer, such as YAP, c-Myc, β-catenin, and ERK [23, 27, 28]. Consequently, by negatively regulating PR55α protein stability, p53 can indirectly inhibit these oncogenic pathways and, at the same time, promote the induction of senescence by removing the inhibition of p16 expression exerted by PR55α. Conversely, the loss of p53 function can result in the induction of PR55α expression [28], which in turn can activate the same group of oncogenic pathways while suppressing the expression of p16 and induction of senescence. Future studies are needed to investigate whether PR55α significantly contributes to the transcriptional suppression of p16 expression in some cancers that harbor mutant p53, such as pancreatic cancer, in which PR55α is coincidently overexpressed [27, 31, 70]. Likewise, when normal human cells become senescent, p53 becomes activated and p16 is induced [47, 71]. These events may also include the downregulation of PR55α by the p53/FBXL20 axis, which could also contribute at least in part to the induction of p16 at senescence. This crosstalk between p53 and the p16/RB pathway enabled by PR55α could potentially explain why in certain normal cells, the loss of p53 function is sufficient to overcome senescence, such as in mouse embryonic fibroblasts [72] or human mammary epithelial cells [73].

Current findings may also be extrapolated to senescence triggered by other genotoxic stressors, such as chemotherapeutic agents. Chemotherapy-induced senescence (CIS) is commonly observed after cancer treatments and is believed to contribute to tumor growth inhibition and cancer remission, whereas emerging evidence suggests that a small subset of tumor cells can escape CIS and lead to cancer recurrence [74, 75]. However, the mechanisms responsible for the bypass of CIS are not well understood. In this report, we show that PR55α overexpression can inhibit the p16/RB pathway and block senescence induction by IR. In future studies, we will determine if PR55α can also block CIS and, in turn, cause chemoresistance in cancer patients.

In response to IR-induced DNA damage, ATM, ATR, and DNA-PK kinases are rapidly activated, which, in turn, induce their respective downstream effectors to activate cell cycle checkpoint responses, leading to cell cycle arrest and DNA repair [50]. However, when DNA damage cannot be repaired, apoptosis or senescence is triggered to eliminate the injured cells [50]. We have not observed evidence indicating that the DNA damage response regulates the level or activity of PR55α-controlled PP2A. But because PR55α inhibits p16 expression, it could instead influence the cells’ decision to undergo cell cycle arrest, senesce, or apoptosis. Future studies will be needed to identify the impact of PR55α on the fate of cellular response to DNA damage and evaluate the significance of this effect on the risk of oncogenic transformation.

During natural aging, there is a gradual accumulation of p16-expressing senescent cells in tissues [76]. To investigate the significance of PR55α in this up-regulation of p16, we compared levels of the p16 and PR55α proteins in a panel of normal tissue specimens derived from young (≤43 y/o) and old (≥68 y/o) donors. The results obtained are consistent with the notion that PR55α negatively regulates p16 transcriptional expression, revealing a significant inverse correlation between p16 and PR55α levels, in young tissues having PR55α-high/p16-low and in older tissue having PR55α-low/p16-high (Figure 8). These results are in line with the notion that PR55α inhibits p16 expression (Figure 1). Future studies will be needed to understand the inter-relationships among p53, FBXL20, PR55α, and p16 expression in human tissues following aging.

p16 expression is commonly regulated at the mRNA level [38]. A few mechanisms are reported to regulate p16, including changes in *CDKN2A* promoter methylation, epigenetic histone marks, altered transcription factors, and changes in the stability of p16 mRNA [77]. Our results indicate that the regulation of p16 expression by PR55α also occurs at the p16 mRNA transcription level. This conclusion is based on our comprehensive analyses of the effects of PR55α on p16 protein stability, p16 mRNA level, and p16 promoter activity (see Figures 3A–3C). Furthermore, since the AUF1 knockdown by siRNA did not prevent the negative regulation of p16 expression by PR55α in HPNE cells (see Figure 3D), AUF1-mediated p16 mRNA decay is also not responsible for the inhibitory effect of PR55α on p16. The *CDKN2A* locus encodes for both p16 and p14 and the entire locus is subjected to epigenetic regulation by polycomb group protein complexes (PcG) and histone modifications (H3K27me3, H2AK119ub, H3K4me3), but the p16 and p14 promoters can still be differentially regulated by their respective transcription factors [78]. Our results show that PR55α specifically reduces p16 expression, not that of p14. While we cannot exclude the possibility that PR55α is also affecting the epigenetic regulators of p16 expression, PR55α-controlled PP2A appears to directly regulate the p16 promoter in luciferase reporter assays (Figure 3). Further studies will be necessary to determine how PR55α regulates p16 transcription and which transcription factors and epigenetic regulators are involved.

Both p53 and p16 are described as key regulators of senescence [1, 8, 43]. p16 inhibits the CDK4/6 kinases and the p53/p21 cascade blocks the CDK2 kinase, both of which result in RB activation and its sequestration of the E2F transcription factor, thus arresting the cell cycle in the G1 phase during senescence [79] (Figure 1). In this study, we assessed the effects of PR55α on the induction of cellular senescence by IR both in normal cells expressing wt p53 (HPNE) and in cancer cells expressing mutant p53 (CD18/HPAF, AsPC-1). Among the three cell lines, only in AsPC-1 cells IR failed to induce senescence (Figure 7) in either the parental control cells (PR55α-high) or PR55α-shRNA knockdown cells (PR55α-low). The main difference among the three cell lines is in the mutational status of their p16 gene. The locus is intact in HPNE cells but is homozygously deleted in the AsPC-1 cells [37, 54] (Figure 7A). In the CD18/HPAF cells, the p16 protein carries an in-frame deletion (aa 29–34) but is still reportedly functional [37, 54]. Our results support the notion that this p16 protein still has retained its ability to induce senescence in response to IR, as its up-regulation by the PR55α knockdown is associated with IR-induced senescence (Figure 6B, 6D). This situation may also apply to other pancreatic cancer cell lines expressing p16 mutants, which may still be biologically functional for inducing cellular senescence. Nonetheless, the current studies cannot exclude the possibility that PR55α may also affect other key regulators of senescence in addition to its inhibition of p16 expression. However, the study of this possibility is beyond the scope of the current investigation.

In summary, the results from this study reveal a novel function of PR55a-controlled PP2A Ser/Thr phosphatase complexes in the inhibition of IR-induced senescence, which involves its inhibition of the p16/RB pathway that inhibits E2F activity to block the cell cycle. Future studies will be needed to identify the precise molecular targets of PR55α within the epigenetic and transcriptional regulators of p16 expression. Together, the results of this report establish PR55α-controlled PP2A phosphatase as a critical new regulator of p16 expression and IR-induced senescence. Future studies will need to delineate the interplays among PR55α, p53, and p16 in the maintenance of cellular homeostasis or induction of cellular senescence, as well as how the dysregulation of these pathways promotes cancer or aging.

## MATERIALS AND METHODS

### Cell culture and treatment

The HPNE cells are primary human pancreatic normal ductal cells immortalized with human telomerase hTERT [32, 33]. The Doxycycline (Dox)-inducible HPNE-PR55α cell line was established by transducing HPNE cells with the pRevTet-On retroviral vector (Clontech Laboratories, Mountain View, CA, USA) modified to express the PR55α cDNA and selecting the cells with hygromycin 200 μg/ml. The ectopic expression of PR55α was induced by 1 μg/ml Dox for 48–72 h. The HPNE isogenic cell lines were maintained in Medium D growth medium (3 parts high glucose Dulbecco’s modified Eagle’s medium (DMEM) (GE Healthcare Life Sciences, Pittsburgh, PA, USA) to one part of M3 medium (INCELL, San Antonio, TX, USA) supplemented with 10% Tet-free FBS and 10 ng/mL human recombinant EGF (Invitrogen, Waltham, MA, USA)) [32, 33]. Human pancreatic cancer cell lines CD18/HPAF and AsPC-1 were obtained from ATCC (Manassas, VA, USA) and maintained in DMEM (Life Technologies, Carlsbad, CA, USA) with 10% fetal bovine serum (FBS) in an atmosphere containing 5% CO_2_. IR exposure was performed by treating exponentially growing cells with a Mark I 68A Cesium-137 Irradiator (JL Shepherd and Associates, San Fernando, CA, USA) at the indicated doses and incubated for the specified times at 37°C.

### Antibodies, Western blot analysis, and protein stability assessment

The primary antibodies used in this study were mouse IgG against p16 (#55079, BD Bioscience, USA), GAPDH (#32233, Santa Cruz, USA), and AUF1 (#12382, Cell Signaling Technologies, USA). Western blot analyses were performed as described previously [23, 28]. In brief, protein samples were separated by SDS-PAGE and transferred to a nitrocellulose membrane. The membrane was then probed with the primary antibody against p16, AUF1, or GAPDH, followed by detection using an anti-mouse IgG secondary antibody conjugated to horseradish peroxidase. Chemiluminescent signals were captured and analyzed with a BioRad Chemidoc System (Hercules, CA, USA). Alternatively, they were revealed after exposure to an x-ray film and scanning of the film with an EPSON Perfection 4490 PHOTO scanner. Signal quantitation was done using the Fiji-ImageJ analytical program (NIH, Bethesda, MD, USA).

To analyze the protein half-life of p16, we exposed cells to protein synthesis inhibitor cycloheximide (CHX, dissolved in water) (Sigma-Aldrich, St. Louis, MO, USA) at 15 μg/ml and we began monitoring the levels of p16 protein by immunoblotting, as described previously [23, 28]. α-Tubulin served as a loading control on the Western blots. The levels of p16 on the blot were normalized by control protein levels, and calculated for half-life times (t(1/2)) by linear regression analysis of log (protein level) against time using the SigmaPlot graphing and data analysis software, as described in our studies [23, 28].

### Senescence-associated β-galactosidase (SA-β-gal) assay

The SA-β-gal activity was measured per the manufacturer’s instructions (Catalog #200488, Agilent Technologies, La Jolla, CA, USA). The SA-β-gal activity was determined by using X-gal (5-bromo-4-chloro-3-indolyl β-D-galactosidase) staining at pH 6.0. Ten randomly selected fields per sample were photographed and quantified for the cells positive in SA-β-gal staining under a light microscope.

### Cell cycle analysis

Cell cycle analysis was carried out as described previously [80, 81]. Briefly, 24 h after IR exposure, the cells were harvested, washed with phosphate-buffered saline (PBS), and fixed in ice-cold ethanol. Cells were stained with propidium iodide (PI) and analyzed for DNA content by fluorescence-activated cell sorting (FACS) analysis using the FACS Calibur instrument (Becton Dickinson, Mansfield, MA, USA). At least 20,000 cells from each sample were evaluated.

### Luciferase reporter constructs and luciferase assay

pGL2-p16-Luc is a firefly luciferase reporter for p16 promoter activity. It carries a firefly luciferase gene under the control of the p16 promoter (from nucleotides −3243 to −165 upstream of the p16 initiation codon). p16 promoter sequences were initially amplified by PCR using primers p16A (5′-cggatatcacgcgtccacccaaggatgccataat-3′; MluI site underlined) and p16B (5′-ctgaagatctcccgccgccggctccat-3′; BglII site underlined). The PCR product was digested with MluI and BglII and inserted into the same sites of the pGL2-Basic vector (Promega, Madison, WI, USA). Next, this plasmid was digested with SacII and BglII and then self-ligated to eliminate the segment −164 to +17 containing the p16 initiation codon. pRL-SV40-Luc (Promega) is a control Renilla luciferase reporter that contains the SV40 promoter. pGL2-p16-Luc and pRL-SV40-Luc were co-transfected into the cells using Lipofectamine 2000 (Invitrogen, Waltham, MA, USA) according to the manufacturer’s suggestions. Firefly luciferase and Renilla luciferase activities were measured at 48 h following transfections using a Luciferase Assay System, as instructed by the manufacturer (Promega). Firefly luciferase activity was normalized for transfection efficiency using the activity of Renilla luciferase.

### RNA isolation and real-time quantitative reverse transcription (qRT)-PCR

RNA was isolated from cells using TRIzol and reverse transcribed using the iScript Reverse Transcription kit. Further, the mRNA was quantified by Real-Time qRT-PCR using SsoAdvanced TM SYBR Green Supermix (Bio-Rad) following the manufacturer’s protocols. GAPDH was used as the internal control. The following primers were used for the PCR: p16 forward primer: 5′-GACCTGGCTGAGGAGCTG-3′ and p16 reverse primer: 5′-CAATCGGGGATGTCTGAGGG-3′; GAPDH forward primer: 5′-TTCCACCCATGGCAAATTCC-3′ and reverse primer: 5′-TGGCAGGTTTTTCTAGACGG-3′.

### Small hairpin RNAs, short interfering RNAs, and transfection

The small hairpin RNAs (shRNA) sequences targeting human PR55α (PPP2R2A) mRNA are 5′-ATGGCTAGCAGACATGGAG-3′, and 5′-CAACTATCTCAACTAAGCA-3′. The non-targeting control shRNA was designed to target no known genes in humans, mice, or rats. The control shRNA sequence is 5′-ATCTCGCTTGGGCGAGAGTAAG-3′ (Dharmacon, Lafayette, CO, USA). The siGENOME/AUF1 short interfering RNAs (siRNAs) SMARTpool (Dharmacon) consists of four siRNAs targeting multiple sites on AUF1 mRNA: 5′-CAAAUUUGGUGAAGUUGUA-3′, 5′-GGAAGGUGAUUGAUCCUAA-3′, 5′-AGACUGCACUCUGAAGUUA-3′, and 5′-CGUGGGUUCUGCUUUAUUA-3′. Control siGENOME nontargeting siRNA (Dharmacon) was designed to target no known genes in humans, mice, or rats. The sequence of the control siRNA is 5′-UAGCGACUAAACACAUCAA-3′. siRNA transfection of cells was performed with DharmaFECT-1 (Thermo Fisher Scientific, Waltham, MA, USA) as instructed by the manufacturer. Transfected cells were incubated for 48–72 hours before analysis.

### Human normal tissue specimens and immunohistochemistry

Human normal tissue samples (CHTN_Norm3 - Normal Tissue Survey) for immunohistochemistry (IHC) analyses were provided by the Cooperative Human Tissue Network (Mid-Atlantic Division) funded by the National Cancer Institute. The normal tissue samples were derived from various human organs/sites including the skin, bladder, breast, digestive tracts, heart, kidney, liver, ovary, pancreas, spleen, and tonsil of both young (≤43 years old) and old (≥68 years old) individuals (Supplementary Table 1). The IHC analysis of PR55α used the anti-PR55α antibody 100C1 (Cell Signaling Technology, Danvers, MA, USA) at the dilution of 1:400. The IHC analysis of p16 used the anti-p16 JC2 mouse monoclonal IgG (ready use, MM156-10, Statlab, USA). The IHC analyses were performed by the Tissue Science Core of the University of Nebraska Medical Center (UNMC). PR55α and p16 IHC staining were evaluated by a UNMC pathologist who was blinded to the clinical information. Immunohistochemistry (IHC) staining was quantified by a Histoscore (H-score) based on both signal intensity (0 = no staining, 1 = weak, 2 = moderate, 3 = strong immunoreactivity) and percentage of positive cells (1 = 0–25%, 2 = 26–50%, 3 = 51–75%, 4 = 76–100%), as we have done in previous studies [27, 28, 82].

### Statistical analysis

Student’s *t*-test and one-way ANOVA methods were used to compare experimental groups using SigmaPlot software (Palo Alto, CA, USA), and the results were expressed as mean ± SD. The statistical analyses were described in the respective figure legends and *P*-values ≤ 0.05 were considered significant.

## Supplementary Materials

Supplementary Table 1
