# Supplementary material for: PR55α-controlled protein phosphatase 2A inhibits p16 expression and blocks cellular senescence induction by γ-irradiation
Source: Aging (Albany NY). 2024 Mar 4;16(5):4116–37. doi: 10.18632/aging.205619 (PMC10968692; doi:10.18632/aging.205619)
Supplement: Supplementary Table 1 [file aging-16-205619-s001.pdf]

SUPPLEMENTARY TABLE

Supplementary Table 1. Human normal tissue samples from various organs/sites of young and old individuals were used to assess PR55α and p16 protein expression by immunohistochemistry with H-score.

Case	Cohort	Tissue	Age	Gender	H-Score PR55α	H-Score p16
1	Young	alveoli	1	Female	4.0	0.0
2	Young	alveoli	1	Female	0.2	0.0
3	Young	mucosa assoc. lymphoid tissue, appendix	6	Male	0.1	0.0
4	Young	mucosa assoc. lymphoid tissue, appendix	6	Male	0.1	0.0
5	Young	alveoli	1	Female	4.5	0.0
6	Young	thymus	19	Female	7.5	1.5
7	Young	thymus	19	Female	4.5	0.9
8	Young	thymus	19	Female	6.0	1.2
9	Young	skin, squamous epithelium	23	Female	0.1	0.2
10	Young	skin, squamous epithelium	23	Female	0.1	0.1
11	Young	skin, squamous epithelium	23	Female	0.2	0.1
12	Young	Breast, epithelium	23	Female	7.5	0.4
13	Young	Breast, epithelium	23	Female	8.0	0.8
14	Young	Breast, epithelium	23	Female	7.0	0.9
15	Young	tonsil, squamous epithelium	24	Female	7.0	0.4
16	Young	tonsil, squamous epithelium	24	Female	9.0	0.8
17	Young	kidney, cortex	25	Male	8.0	0.2
18	Young	kidney, medulla	25	Male	7.5	0.1
19	Young	kidney, medulla	25	Male	1.5	0.1
20	Young	ectocervix	26	Female	0.2	1.5
21	Young	Smooth muscle, uterus	28	Female	3.0	0.3
22	Young	Smooth muscle, uterus	28	Female	2.0	0.0
23	Young	Smooth muscle, uterus	28	Female	3.0	0.3
24	Young	Ovary, 1' oocytes	33	Female	9.0	0.3
25	Young	Ovary, 1' oocytes	33	Female	0.0	0.2
26	Young	Ovary, 1' oocytes	33	Female	0.2	0.2
27	Young	ovary, corpus luteum	33	Female	6.5	0.9
28	Young	ovary, corpus luteum	33	Female	7.5	1.5
29	Young	ovary, corpus luteum	33	Female	8.0	0.9
30	Young	endocervix	37	Female	1.0	0.0
31	Young	endocervix	37	Female	0.2	0.0
32	Young	amniotic membrane	39	Female	4.0	0.0
33	Young	amniotic membrane	39	Female	2.0	0.2
34	Young	amniotic membrane	39	Female	9.0	0.9
35	Young	fallopian tube	43	Female	10.0	0.3
36	Young	fallopian tube	43	Female	13.0	0.2
37	Young	fallopian tube	43	Female	2.0	0.0
38	Old	thyroid	68	Female	0.2	0.0
39	Old	thyroid	68	Female	0.0	0.1
40	Old	adrenal gland, cortex	71	Male	8.0	0.1
41	Old	adrenal gland, cortex	71	Male	8.0	0.0
42	Old	adrenal gland, medulla	71	Male	10.0	0.1
43	Old	adrenal gland, medulla	71	Male	7.0	0.1

44	Old	adrenal gland, medulla	71	Male	4.0	0.0
45	Old	esophagus, squamous mucosa	74	Female	4.5	0.1
46	Old	esophagus, squamous mucosa	74	Female	6.0	0.3
47	Old	esophagus, squamous mucosa	74	Female	5.0	3.0
48	Old	peripheral nerve	73	Female	0.4	0.1
49	Old	peripheral nerve	73	Female	0.4	0.0
50	Old	peripheral nerve	73	Female	0.4	0.0
51	Old	cartilage, articular	73	Female	0.8	0.0
52	Old	aorta, smooth muscle	85	Female	0.1	0.0

The “Young” cohort was ≤43 years old; the “Old” cohort was ≥68 years old.
